# Ecological Pain and Physical Activity Volume and Patterns in Older Adults

**DOI:** 10.1093/geroni/igaf122.3371

**Published:** 2025-12-31

**Authors:** Yurun Cai, Paul Scott, Julia Hooker, Ann Barney, Sophia Holena

**Affiliations:** University of Pittsburgh, Pittsburgh, Pennsylvania, United States; University of Pittsburgh, Pittsburgh, Pennsylvania, United States; University of Pittsburgh, Pittsburgh, Pennsylvania, United States; University of Pittsburgh, Pittsburgh, Pennsylvania, United States; University of Pittsburgh, Pittsburgh, Pennsylvania, United States

## Abstract

Chronic pain is associated with lower physical activity (PA) levels in older adults. However, pain characteristics change over time, and the impacts of changes in pain levels on daily PA quantities and patterns remain unknown. The PRIME pilot study recruited 30 older adults (mean age=74.1±6.0y) with multisite pain (≥2 pain sites in shoulder, elbow, hand/wrist, back, hip, knee, or ankle/foot) in Pittsburgh and surrounding areas in Pennsylvania. Pain characteristics (e.g., severity, laterality, interference) were measured using questionnaires during the 2-hour clinic visit. After the clinic visit, ecological pain was assessed using Qualtrics surveys four times a day, along with Actigraph accelerometer assessment on the non-dominant wrist for 14 days. Linear regression models showed that participants with back pain had significantly lower activity counts (β=-493,271, p = 0.020), higher activity fragmentation (β = 5.0%, p = 0.005), and fewer active minutes (β=-88.4, p = 0.017). Number of pain sites and severity of hand/wrist pain decreased over a day (β=-0.08, p = 0.019; β=-0.09, p = 0.042, respectively) but tended to be stable across 14 days. Linear mixed models showed that every one-unit higher in overall pain severity at one time point was associated with 28,480 fewer activity counts at the subsequent time point (p = 0.012), and these associations were weaker at later time points over a day (p = 0.011). The severity and distribution of pain vary throughout the day, and the impact of pain severity on daily activity weakens as the day progresses. Individualized pain management programs should be provided to older adults with varying pain characteristics, particularly early in the day.

